# P01.26. Traditional Chinese herbal formula HLXL controls ongoing arthritis by regulating the immune mediators of leukocyte trafficking and joint damage

**DOI:** 10.1186/1472-6882-12-S1-P26

**Published:** 2012-06-12

**Authors:** S Nanjundaiah, D Lee, Z Ma, H Yu, L Lao, H Fong, B Berman, K Moudgil, B Astry

**Affiliations:** 1 McLean Hospital, Harvard Medical School, Belmont, USA; 2 Center for Integrative Medicine, Univ of Maryland School of Medicine, Baltimore, USA; 3 Medicinal Chemistry and Pharmacognosy, Univ. of Illinois at Chicago, Chicago, USA; 4 University of Maryland School of Medicine, Baltimore, USA

## Purpose

Rheumatoid arthritis (RA) is a debilitating autoimmune disease characterized by chronic inflammation of the synovial joints. Because of the high cost and adverse effects of the conventional anti-arthritic agents, there is an increasing trend towards using complementary and alternative medicine (CAM) products, including traditional Chinese medicine (TCM). Huo-luo-xiao-ling dan (HLXL) is a TCM herbal formula consisting of 4 herbs that has been used in China for centuries for the treatment of rheumatic diseases. We have modified the traditional herbal formula by adding 7 herbs. The objective of this study was to determine the mechanism of action of modified HLXL (mHLXL) using the rat adjuvant arthritis (AA) model of RA.

## Methods

Arthritis was induced in the Lewis rat by subcutaneous immunization with heat-killed M. tuberculosis H37Ra (Mtb). Beginning at the onset of arthritis, rats were treated either with mHLXL (2.3g/Kg/d by gavage) or with the vehicle (water) and then continued throughout the disease course. At day 18 after disease induction, rats were sacrificed and their draining lymph node cells, synovium-infiltrating cells and sera were harvested for testing. Specific cytokines, chemokines and antibodies were tested using the appropriate methods. The results of the experimental and control groups were statistically analyzed and compared.

## Results

Rats treated with mHLXL showed significantly reduced severity of arthritis compared to the control rats. The suppression of arthritis was associated with a reduction in the levels of pro-inflammatory and bone-damaging cytokines (e.g., IL-17, IL-1, and RANKL) as well as antibodies against the disease-related antigen, heat-shock protein-65. Also reduced were the levels of chemokines (e.g., RANTES) that play a vital role in leukocyte trafficking into the joints in AA and RA.

## Conclusion

Thus, mHLXL is a promising anti-arthritic TCM formula that mediates its effect via downregulating the pro-inflammatory and osteoclastogenic cytokines and chemokines. Our results warrant that mHLXL be evaluated in RA patients.

